# Research on an Improved YOLOv11-Based Detection Method for Harvestable Safflower Filaments in Unstructured Environments

**DOI:** 10.3390/s26154727

**Published:** 2026-07-25

**Authors:** Lingfang Chen, Bangbang Chen, Liqiang Wang, Xiangdong Liu, Baojian Ma

**Affiliations:** School of Mechatronic Engineering, Xinjiang Institute of Technology, Aksu 843100, China; 2020031@xjit.edu.cn (L.C.); 2019049@xjit.edu.cn (L.W.); 2017011@xjit.edu.cn (X.L.); 11813019@zju.edu.cn (B.M.)

**Keywords:** red flower detection, YOLOv11n, Starnet network, ADown module, lightweight model

## Abstract

To address the demands of intelligent safflower harvesting scenarios, which require a safflower recognition model with both low computational cost and high detection performance, this paper proposes a lightweight improved model based on YOLOv11n, termed YOLOv11n-Starnet-ADown. To reduce the model’s size, the Starnet network is adopted to replace the backbone network of YOLOv11n. To enhance small-object detection capability while further reducing memory footprint, ADown is used to replace the standard convolutional downsampling module in the neck network of YOLOv11n. The YOLOv11n-Starnet-ADown model was experimentally validated on a self-constructed safflower dataset. The results show that the model achieves an overall precision of 91.6%, a recall of 87.8%, and an mAP@0.5 of 92%; the recognition accuracy for harvestable safflower filaments reaches 96.5%; and the model memory footprint is 3.66 MB, representing a 29.9% reduction compared to the baseline YOLOv11n model. Finally, the detection performance of YOLOv11n-Starnet-ADown was compared with that of four conventional models under different scenarios, confirming the effectiveness of the proposed model. The proposed model exhibits stable detection performance under diverse complex conditions, including overcast skies, occlusion, and backlighting, which adequately satisfies the fundamental requirements for safflower filament detection in real-world environments. Overall, this work offers a lightweight technical solution for the intelligent harvesting of safflower filaments in unstructured settings.

## 1. Introduction

Safflower is widely used in medicine, textile dyeing, and other fields, making it a crop of considerable economic value [1]. In safflower cultivation, because the maturity of safflower filaments varies greatly and the flowering period is short, harvesting relies mainly on manual, multiple-pass picking. This harvesting method is characterized by low efficiency, high labor intensity, and high labor costs, which hinder the large-scale and intelligent development of the safflower industry. To improve harvesting efficiency and reduce production costs, intelligent recognition and mechanized picking have become the development trends for safflower cultivation.

Existing technologies still face numerous challenges in safflower recognition, especially in real, densely planted, unstructured environments. Owing to variable field lighting, weed occlusion, overlapping branches and leaves, and complex backgrounds, target misses and false detections occur very easily. Moreover, the maturity of safflower filaments varies significantly; the three target classes—mature filaments, immature filaments, and buds—exhibit high morphological similarity and tiny dimensions, making fine-grained classification detection extremely difficult. This poses substantial challenges to both detection accuracy and efficiency. Harvesting robots are embedded terminal devices with limited hardware computing power and storage resources. Conventional high-accuracy detection models have a large model size and high inference latency, failing to meet the demands of real-time field detection. Meanwhile, common lightweight models generally suffer from accuracy degradation and insufficient feature extraction capability, making it difficult to balance lightweight design with high detection accuracy. Therefore, achieving both a small model size and good detection performance presents a dual technical challenge for safflower filament detection models.

YOLO (You Only Look Once), with its end-to-end architecture, fast inference speed, and concise structure, has become one of the mainstream frameworks for agricultural detection. Many researchers have applied YOLO models to intelligent recognition in safflower harvesting. Guo et al. [2] proposed an improved algorithm, SF-YOLO, based on YOLOv5 to enhance model robustness under varying lighting, image noise, and diverse viewing angles. Compared with the original YOLOv5s model, the computational cost (GFLOPs) was reduced from 15.8 G to 13.2 G, and the number of parameters (Params) dropped from 7.013 M to 5.34 M, corresponding to reductions of 16.6% and 23.9%, respectively. Meanwhile, the mAP@0.5 metric improved by 1.3 percentage points to 95.3%, achieving both a significant reduction in memory footprint and an increase in mean average precision. Ma et al. [3] constructed a coarse-to-fine two-stage classification framework and designed a lightweight hybrid network, CNATNet, based on convolution and attention mechanisms, improving inference speed while ensuring recognition accuracy. Chen et al. [4] addressed the difficulty safflower harvesting robots face in accurately detecting and locating picking points in unstructured planting environments. They proposed a detection-segmentation-OpenCV-extraction (DSOE) fusion method based on an improved YOLO model, built a localization system with a depth camera, and finally integrated a Delta robotic arm with a depth camera to construct a positioning control system that computes the three-dimensional spatial coordinates of picking points. Duan et al. [5] addressed issues such as cluttered field backgrounds, variable viewing angles, and unstable lighting conditions, which often lead to missed detections, false positives, and imbalanced distribution of small targets for safflower picking robots. They embedded a VSS-SPPF module in the backbone, adopted an AFPN structure in the neck, and introduced a self-supervised super-resolution auxiliary branch (SRSS) to propose the SAF-YOLO detection model. This model achieved a precision of 90.1%, recall of 85.9%, and mAP of 93.3%. Zhang et al. [6] proposed an improved Faster R-CNN detection model to solve the challenges of safflower filament detection under different lighting conditions, branch and leaf occlusion, and complex weather. They adopted a ResNeSt-101 residual network, ROI Align, and the partitioning around medoids (PAM) clustering algorithm to enhance small target detection accuracy, achieving an mAP of 91.49% for precise safflower filament detection. Zhang et al. [7] proposed a method for locating safflower picking points throughout the full harvest period based on the SBP-YOLOv8s-seg network. By optimizing the detection and segmentation network, they improved accuracy, raising the precision, recall, and mAP from 87.9%, 79%, and 84.4% of the original YOLOv8s-seg to 89.1%, 79.7%, and 85.7%, respectively. Chen et al. [8] adopted MobileNetv2 as the backbone and integrated depthwise separable convolution (DSC) and coordinate attention (CA) mechanisms to improve YOLOv5s, proposing the YOLOv5s-MCD model. Compared with the original YOLOv5s, the model size was reduced by 7.69 MB, the mAP reached 95.6%, and the average inference time per image was only 3.2 ms, enabling rapid and accurate real-time detection of safflower filaments. Existing algorithms have taken both detection performance and recognition efficiency into account, but there is still considerable room for improvement to meet the demands of real-time detection.

Although YOLO models demonstrate good detection performance, their relatively large parameter scales still limit their application on agricultural edge computing devices. Many researchers have investigated lightweight improvements to YOLO models for specific scenarios. Zhu et al. [9] built a lightweight detection model, Lib-YOLO, with Starnet as the backbone, suitable for book detection and text recognition on embedded edge devices. The Lib-YOLO model has only 1.39 M parameters and achieves an mAP as high as 99%, balancing real-time performance and recognition accuracy. Ji et al. [10] replaced the native CSPDarknet53 backbone with the Starnet architecture within the YOLOv8n framework, proposing a lightweight detection algorithm called SCL-YOLOv8. The model size is only 3.1 MB, and compared with YOLOv8n, SCL-YOLOv8 attains an mAP@0.5 of 94.2%, reduces the parameter count by 56.8%, cuts floating-point operations (FLOPs) by 45.7%, and shrinks the model file size by 50%. Zeng et al. [11] used YOLOv9-C as the baseline and embedded six mainstream backbones—ResNet50, GhostNet, MobileNetV4, FasterNet, Starnet, and RepViT—into the YOLOv9 framework, conducting cross-validation on the YOLOv5-m model and systematic comparative experiments on two public steel defect datasets, NEU-DET and GC10-DET. Their work demonstrated the advantages of lightweight networks in industrial real-time inspection scenarios. Du et al. [12] proposed an improved algorithm, YOLOv11n-sps, which optimized the YOLOv11n backbone with a lightweight Starnet network, combined with depthwise separable convolution and channel shuffling mechanisms, to achieve precise defect identification in photovoltaic modules. Compared with the original YOLOv11n, the model size was reduced by 1.23 MB. Pang et al. [13] integrated the Starnet backbone with a bidirectional feature pyramid network (BiFPN) into YOLOv10n, proposing the SNBF-YOLO model. Relative to the baseline YOLOv10n, this model improved precision, recall, and mAP for railway track damage detection by 19.4%, 13.2%, and 14.3%, respectively, while the parameter count was only 1.64 MB. Chang et al. [14] proposed an improved SAG-YOLO algorithm for chick sex detection, replacing the original feature extraction backbone with a lightweight Starnet network. Compared with the baseline YOLOv10n, they achieved improvements of 1.3%, 2.6%, and 1.5% in precision, recall, and mAP, respectively, while reducing the parameter count by 0.8633 MB. He et al. [15] designed a lightweight SAR ship detection algorithm, SSGY, whose backbone incorporates a Starnet structure integrating multi-scale convolution kernels, dilated convolutions, and an efficient channel attention (ECA) module, reducing the parameter count to 1.4 MB while slightly improving detection accuracy. Chen et al. [16] adopted the Starnet architecture as the backbone for YOLOv8 and made lightweight improvements to the C2f module and the detection head. They developed a lightweight shared-convolution detection head, Detect_EL, and upgraded the CIoU loss to the PIoUv2 loss function, proposing a lightweight YOLO-SaFi model. Compared with the original YOLOv8, YOLO-SaFi reduces the parameter count by 50.0%, computational load by 40.7%, and model weight file size by 48.2%; meanwhile, recall is improved by 1.9%, mAP by 0.3%, and inference frame rate increases by 88.4 FPS.

Because safflower filament maturity varies greatly, the flowering period is short, bud sizes differ significantly, and the distance from the camera lens to the target filaments changes, some detection targets appear relatively small in the entire image. Immature safflower filaments, in particular, are small and difficult to detect [2]. Therefore, enhancing the small-target recognition capability of safflower identification models is crucial. Gao et al. [17] replaced the conventional convolution modules in the YOLOv11n backbone with an asymmetric downsampling module (ADown), embedded a parameter-free SimAM attention mechanism, and adopted a lightweight and efficient BiFPN structure to reconstruct the original feature fusion layer, proposing a lightweight single-stage object detection algorithm named Garlic-YOLO-DD. Compared with the original YOLOv11n, in garlic damage detection, Garlic-YOLO-DD reduced the parameter count to only 57.96% of the baseline, cut computational load by 20.63%, increased inference speed by 15.97%, and improved the mean average precision at IoU threshold 0.5 (mAP@0.5) by 27.64%. Wang et al. [18] built upon YOLOv11n by integrating the C3Ghost module for efficient feature fusion and adopting the ADown module in place of conventional convolution downsampling, proposing an optimized model, ADG-YOLO, suitable for real-time target detection and ranging on UAV platforms. This model reduced the parameter count from 2.58 MB to 1.77 MB. Tang et al. [19] addressed the detection of tomato early blight by embedding the C3k2_iAFF attention fusion module into YOLOv11n and introducing the ADown multi-branch downsampling structure, resulting in a lightweight detection model named YOLOv11-AIU. Compared with YOLOv11n, the model size was reduced from 5.5 MB to 4.7 MB. Tian et al. [20] tackled the challenge of precise real-time instance segmentation of cabbage heads in unmanned cabbage harvesting scenarios by proposing an improved YOLOv8n-seg instance segmentation network. They introduced a deformable attention mechanism with dynamic sampling points, added the ADown lightweight module, and designed a small-target enhancement pyramid network based on the PAFPN structure. Their model achieved a precision of 92.2%, recall of 87.2%, and mAP@0.5 of 95.1%, with a model file size of only 6.46 MB.

Existing safflower detection models are primarily improved upon YOLOv5, YOLOv8, and similar architectures, with modifications mainly focusing on specific layers, replacement of certain components, the introduction of attention mechanisms, or the adoption of lightweight backbones such as MobileNet or Starnet. Numerous lightweight YOLO variants have been proposed for crop recognition and have achieved satisfactory performance in various scenarios. However, existing lightweight YOLO models either simply replace the backbone with Starnet or only optimize the neck structure; none have jointly applied Starnet and ADown to agricultural small-target detection, nor have they conducted task-oriented fine-tuning of Starnet’s depth parameters. Moreover, research on lightweight models specifically tailored for safflower filament recognition remains scarce. Based on the YOLOv11n baseline model, this paper carries out model lightweighting and performance optimization, proposing a lightweight model for safflower filament detection called YOLOv11n-Starnet-ADown. By constructing a dedicated multi-class safflower filament dataset, screening multiple variant models, introducing the lightweight Starnet backbone to replace the original backbone, optimizing the neck downsampling structure with the ADown module, fixing the total network depth, and finely adjusting the Starnet depth configuration parameters, a high-accuracy lightweight model suitable for safflower filament detection was ultimately obtained. Through ablation experiments and comprehensive multi-model comparisons across various scenarios, we thoroughly validate the effectiveness of our improvement strategies. The results demonstrate that the improved model achieves a significant reduction in model size while maintaining or even exceeding the detection performance of the baseline YOLOv11n, particularly exhibiting excellent recognition capability for harvestable mature safflower filaments. This work can provide reliable algorithmic support for real-time field detection and intelligent harvesting by safflower picking robots.

## 2. Dataset Construction and Model Development

### 2.1. Safflower Image Data Acquisition

The original images used in the dataset were captured in the experimental field of Xinjiang Institute of Technology in Wensu County, Aksu Prefecture, Xinjiang. The shooting environment was a real safflower planting environment. Images were taken under various lighting conditions, different planting densities, and different plant maturity stages. The shooting device was the built-in camera of a Redmi K70 mobile phone, with original image resolutions of 3456 × 4608 and 1280 × 1706 pixels.

Safflower images were taken from 1 August to 2 October 2023, encompassing different weather conditions, time periods, angles, and planting densities. They cover various natural lighting scenarios such as sunny, cloudy, backlit, and side-lit conditions, as well as different times of day including morning, noon, and evening, simulating the all-day operating environment of field robots. Based on the actual working height of the harvesting robot, the shooting angles include horizontal, top-down, and oblique views, fully reproducing real field operation perspectives. Complex field scenarios such as sparse and dense plants, branch and leaf occlusion, weed interference, excessive light, and insufficient light are also included, maximizing the scene diversity of the dataset to prevent model overfitting and poor generalization. A total of 1470 original images were obtained.

**Figure 1:**
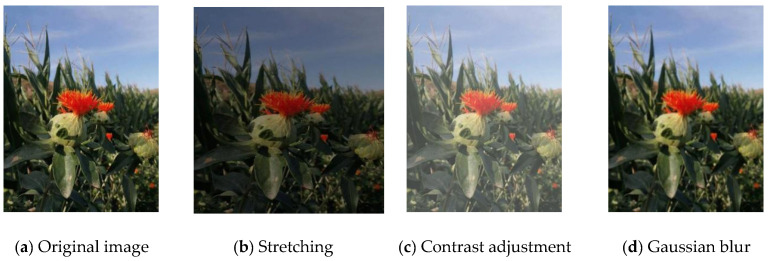
Examples of safflower images.

To achieve better detection performance in various environments and improve the robustness and generalization ability of the system, data augmentation was applied to some images, including stretching, contrast adjustment, Gaussian blurring, and image resizing. This resulted in a final dataset of 3990 images. Examples of the safflower image augmentation effects are shown in Figure 1.

### 2.2. Safflower Dataset Construction and Splitting

The open-source labeling software LabelImg 1.8.6 was used to manually annotate the augmented image set. Three classes of labels were defined: mature safflower filaments were labeled as mature, immature safflower filaments as immature, and flower buds as bud. Table 1 presents the count statistics for each label class. The number of immature labels is 1156, the number of mature labels is 12,306, and the number of bud labels is 29,021. A class imbalance exists in the dataset; in particular, the number of immature labels is relatively low, which may cause the model to learn this class poorly during training. Therefore, subsequent model optimization needs to account for this situation in order to mitigate the impact of the limited number of immature labels on detection accuracy.

To ensure the scientific validity and objectivity of model training, validation, and testing, the dataset was randomly split in a 7:2:1 ratio, yielding 2793 training images, 798 validation images, and 399 test images. The training set is used for iterative model parameter updates and feature learning, the validation set is used for monitoring model performance and fine-tuning hyperparameters during training, and the test set is used for final model performance evaluation and generalization ability verification. The three sample subsets exhibit uniform scene distribution and balanced class quantities without sample skew issues, laying a data foundation for stable model training and performance evaluation.

## 3. Improved YOLOv11n Model for Safflower Recognition

This paper adopts YOLOv11n as the baseline model. YOLOv11 is a new-generation YOLO series detection model introduced by the Ultralytics team. Compared with previous models such as YOLOv8, YOLOv9, and YOLOv10, it has undergone comprehensive optimization in network structure, feature fusion, loss function, and training strategy, achieving an integrated upgrade in detection accuracy, inference speed, and model lightweighting. YOLOv11n (nano) is the most lightweight version, specifically designed for low-compute-power devices such as embedded systems, mobile terminals, and robotic terminals. Compared with standard-sized models, YOLOv11n significantly reduces memory footprint and computational cost by streamlining convolution channels, reducing the number of stacked network layers, and optimizing residual structures, while retaining core feature extraction and multi-scale detection capabilities. It is the optimal baseline model choice for real-time detection tasks on agricultural robots [21]. In the context of unstructured environments for safflower harvesting, the YOLOv11n model is well suited for detection scenarios in safflower picking.

### 3.1. YOLOv11n Network Architecture

The overall network architecture of YOLOv11n is divided into three parts: the backbone, the neck, and the head. The backbone is responsible for extracting low-level, mid-level, and high-level features from images and completing feature encoding of the input image. The neck adopts a multi-scale feature fusion structure to achieve concatenation, fusion, and enhancement of features from different levels, compensating for the loss of shallow feature details and the lack of semantic information in deep features. The detection head uses a lightweight decoupled head structure to perform object classification and bounding box regression separately, improving detection accuracy and inference speed.

**Figure 2:**
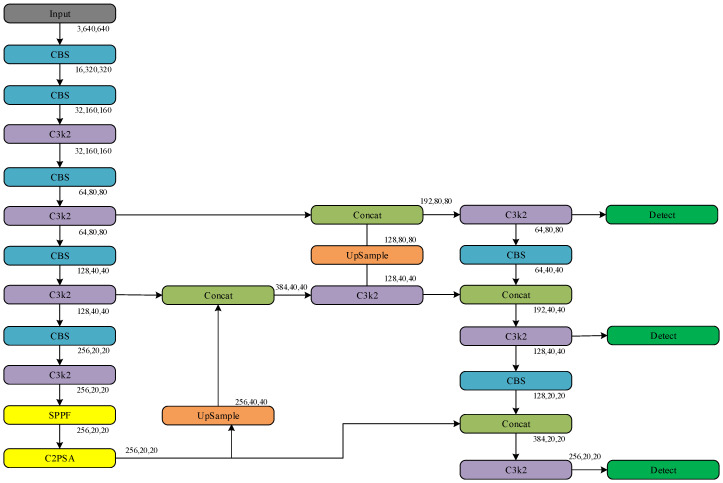
Structure of the YOLOv11n model.

Compared with classic lightweight models such as YOLOv8n, the native YOLOv11n optimizes the C3k2 residual module structure and improves the gradient flow mechanism, effectively alleviating the problem of weak feature extraction capability caused by insufficient depth in lightweight networks. It also optimizes the positive–negative sample matching strategy, enhancing the detection performance for small and dense objects, making it more suitable for detection scenarios involving tiny safflower filaments in dense distributions. However, under complex field background interference, the native YOLOv11n still suffers from issues such as weak fine-grained feature discrimination, loss of small-object features during downsampling, and inadequate adaptability of network parameter configuration. Targeted lightweight improvements are required to further balance model accuracy and model size [22]. Figure 2 shows the structure of the YOLOv11n model.

### 3.2. Baseline Model Screening

Using the YOLOv11n model as the benchmark, five variant models with different existing structures were constructed in order to screen for the baseline model that is best suited to the safflower filament detection task and possesses optimal potential for lightweight improvement. The optimal baseline model was identified through training with unified experimental parameters and performance comparison. The variant models that participated in the screening included YOLOv11n-C3k2-Faster, YOLOv11n-C3k2-Star, YOLOv11n-ADown, YOLOv11n-bifpn, and YOLOv11n-Starnet.

YOLOv11n-C3k2-Faster optimizes the convolution operation logic of the C3k2 module, simplifies the computation process, reduces model inference latency, and improves detection speed [23], but feature extraction accuracy suffers a slight degradation. YOLOv11n-C3k2-Star embeds the StarBlock module from the Starnet network into the C3k2 residual structure, leveraging the lightweight feature enhancement advantages of the StarBlock module to boost feature extraction capability while maintaining a low memory footprint, making it suitable for lightweight detection scenarios [24]. YOLOv11n-ADown replaces the native convolution downsampling with the ADown dynamic downsampling module, addressing the problems of feature loss and detail destruction in traditional downsampling, improving small-object detection accuracy, and offering significant lightweight advantages [25]. YOLOv11n-bifpn introduces a bidirectional feature pyramid network to replace the native neck feature fusion structure, enhancing bidirectional multi-scale feature fusion capability and improving feature utilization in complex scenes; however, structural complexity and memory footprint increase markedly, which is not conducive to lightweight deployment [26]. YOLOv11n-Starnet employs the lightweight Starnet_s050 network to replace a portion of the backbone structure, substantially streamlining network parameters while retaining excellent feature extraction performance, representing an extremely lightweight improvement approach.

All models were trained uniformly for 300 epochs, and core metrics such as overall precision, recall, mAP@0.5, and memory footprint were recorded. The comprehensive performance of each model was compared and analyzed. The detection performance of each model is shown in Table 2. As shown in Table 2, all models can achieve relatively good detection performance on the safflower dataset, but none exhibit breakthrough improvements in both accuracy and speed. The two improved models, YOLOv11n-Starnet and YOLOv11n-ADown, demonstrated the best performance in terms of model size and overall detection performance, respectively, and were therefore selected as the baseline models.

### 3.3. Starnet Lightweight Backbone Network

To enable the model to be deployed on embedded edge computing hardware used in field operations, the Starnet network was selected as the feature extraction network. This can significantly reduce the parameter scale of the entire recognition model and decrease the computational cost of model inference. Starnet is a lightweight neural network backbone architecture [27]. Its four-stage feature extraction architecture follows a progressive paradigm of stepwise channel increase and stepwise feature map size compression. After the input image passes through an initial convolution, it enters Stage1 with channel number C and spatial dimensions H × W. In subsequent stages, convolutional downsampling and multiple StarBlock operations achieve a stepwise channel expansion from *d* to 4d, while the feature map size is halved layer by layer from *H/2* × *W/2* down to *H/32* × *W/32*. This design, with low channel count and high resolution in shallow layers and high channel count and low resolution in deep layers, achieves a precise trade-off between computational cost and feature representation capability in lightweight networks.

StarBlock is the core feature enhancement unit of Starnet. It first performs preliminary feature extraction on the input through a depthwise convolution (DWConv), then stabilizes the feature distribution and accelerates training convergence via batch normalization (BN). The features are subsequently fed into two parallel fully connected layers for linear transformation, with one path using the ReLU6 activation function to limit activation values to within 6. The two outputs undergo feature fusion through the star operation, after which another DWConv layer completes high-level feature extraction and dimension compression. Finally, the result is output through a residual connection with the original input, ensuring training stability.

**Figure 3:**
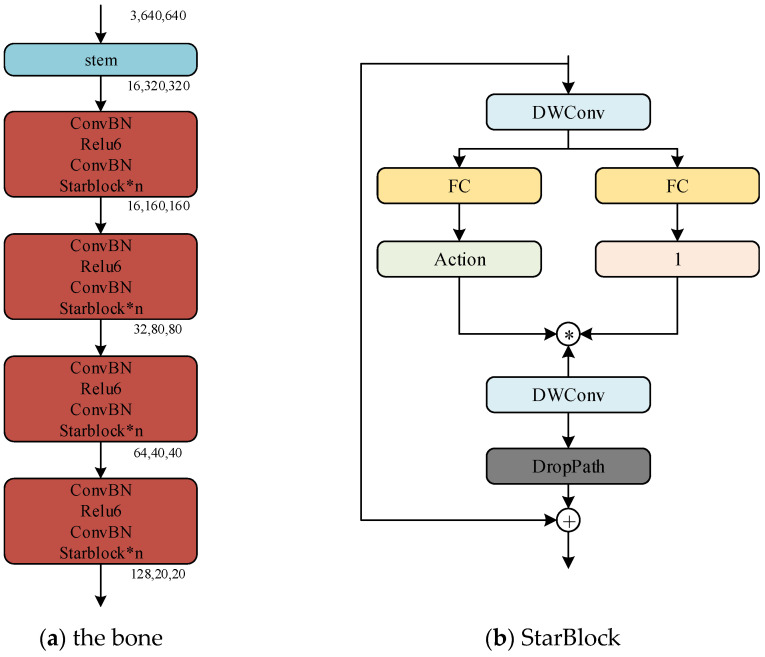
Architecture of the Starnet.

The star operation is the core innovation of Starnet. After stacking multiple layers, the feature dimensions can grow exponentially, achieving high-dimensional feature representation at an extremely low computational cost. Computations are completed in a low-dimensional space while generating high-dimensional features, balancing computational efficiency and representational capacity, and simultaneously capturing both spatial and channel-wise information. The formula of StarBlock is as follows:

(1) $$X^{\left(0\right)}={\mathrm{DWConv}}_{7\times 7}(X_{\mathrm{in}})$$

(2) $$X_{1},\;X_{2}={{\mathrm{Conv}}_{1\times 1}}^{\left(1\right)}(X^{\left(0\right)}),\;{{\mathrm{Conv}}_{1\times 1}}^{\left(2\right)}(X^{\left(0\right)})$$

(3) $$X^{\left(1\right)}=\mathrm{Relu}6(X_{1})\cdot X_{2}$$

(4) $$X_{\mathrm{out}}=X_{\mathrm{in}}+\mathrm{DropPath}({\mathrm{DWConv}}_{1\times 1}(X^{\left(1\right)}))$$

where $X_{\mathrm{in}}$ is the input, $X^{\left(0\right)}$ is the result of the depthwise separable convolution, $X_{1}$ and $X_{2}$ are the outputs of the star branches, $X^{\left(1\right)}$ is the result of the star operation, $X_{\mathrm{out}}$ is the output of the StarBlock module, DWConv is the depthwise separable convolution, Conv is the convolution, Relu6 is the activation function, “·” denotes the star operation. Figure 3 shows the architecture of the Starnet.

### 3.4. ADown Downsampling Module

The neck network of YOLOv11n follows the traditional feature pyramid fusion architecture. During multi-scale feature concatenation and downsampling dimension transformation, standard convolution downsampling with a fixed stride is used throughout to compress feature map size and align channels. This conventional downsampling approach is a rigid sampling mechanism with fixed sampling rules and poor adaptive capability; it can only mechanically accomplish the basic task of feature dimension reduction without the ability to dynamically adjust sampling weights according to image object distribution and feature importance. In the scenario of detecting tiny safflower filament targets in the field, this approach has significant drawbacks, severely constraining the improvement of model detection performance.

**Figure 4:**
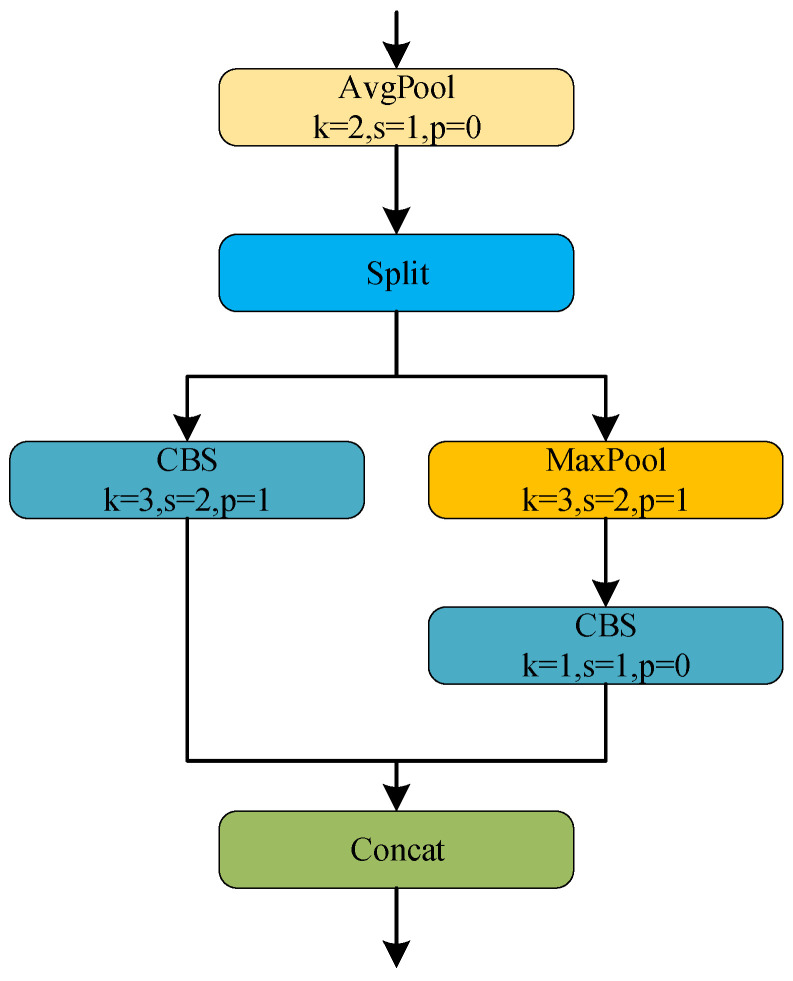
Structure of the ADown module.

The improved neck network replaces convolution downsampling by incorporating ADown. The ADown module is a convolutional block designed for downsampling operations in object detection tasks. In deep learning models, downsampling is a common technique used to reduce the spatial dimensions of feature maps, helping the model capture image features at higher levels while reducing computational cost. Reference [25] introduces the ADown module to perform this operation in an efficient manner with minimal impact on performance. It overcomes the drawbacks of conventional pooling methods, such as max pooling, which suffer from severe information loss and are unfavorable for small targets, thereby enhancing the detection capability for minor defects on solar panels. In this paper, we likewise adopt the ADown module to improve the model’s performance in detecting small targets; the use of strided convolutions improves the computational efficiency of the model but may overlook local details. Overall, the ADown module achieves a balance between information retention and computational efficiency. The structure of the ADown module is shown in Figure 4.

The ADown module first performs average pooling preprocessing:

(5) $$X_{\mathrm{avg}}=Avg{\mathrm{Pool}}_{2\times 2}(X)$$

Second, channel splitting:

(6) $$X_{1},X_{2}=Split(X_{avg},\dim=1)$$

In path 1, standard convolution is used for downsampling:

(7) $$Y_{1}=\mathrm{SiLU}\{BN[Conv_{3\times 3}(X_{1},stride=2,padding=1)]\}$$

In path 2, after max pooling reduces the dimensionality, a 1 × 1 standard convolution is applied for downsampling:

(8) $$Y_{2}=\mathrm{SiLU}\{BN[Conv_{1\times 1}({\mathrm{MaxPool}}_{3\times 3}(X_{2},stride=2,padding=1))]\}$$

Finally, the features from path 1 and path 2 are fused:

(9) $$Y={\mathrm{Concat}([Y}_{1}{,Y}_{2}],\dim=1)$$

In the above, X is the input feature map, Y is the output of the ADown module, AvgPool denotes the average pooling layer, Split denotes channel splitting, $X_{1},X_{2}$ are the results of channel splitting, $Y_{1}$ and $Y_{2}$ are the outputs of the branches, dim is the feature map dimension, Conv denotes the convolution operation, *BN* denotes batch normalization, *SiLU* is the activation function, MaxPool denotes the Maximization pooling layer, Concat is concatenation along dimension.

By reducing the memory footprint, the ADown module decreases model complexity. This helps improve the model’s operational efficiency, especially in resource-constrained environments, making it suitable for the edge computing conditions required for the intelligent recognition of safflower filaments described in this paper.

### 3.5. Optimization of Starnet Backbone Network Parameters

On the basis of completing the replacement of the backbone with Starnet_s050 and the optimization of the neck structure with ADown, the overall lightweight level and feature extraction capability of the model have been significantly improved. However, the fixed network stacking depth parameters cannot be fully adapted to the specific detection characteristics of tiny safflower filament targets, fine-grained classification, and complex field interference. For lightweight deep learning networks, the overall stacking depth and the module allocation ratio at each stage directly determine the model’s feature learning emphasis: shallow layers are responsible for extracting detailed features such as texture, edges, and color; middle layers are responsible for feature fusion, object structure modeling, and discriminative feature enhancement; and deep layers are responsible for global semantic feature extraction and object classification logic construction. The Starnet_s050 network with fixed default parameters is designed for general vision tasks, with balanced feature learning weights and broad generalization. However, for the three highly similar fine-grained safflower filament classes, there are problems such as unreasonable feature learning allocation, insufficient feature extraction at critical layers, and parameter redundancy at non-critical layers, resulting in the model’s accuracy failing to reach the optimal upper limit. Therefore, on the premise of strictly fixing the total network stacking depth unchanged, without increasing the model memory footprint and computational cost, and without disrupting the overall lightweight structure, this paper conducts fine-grained comparative debugging and specialized optimization of the four-stage depth configuration parameters of Starnet_s050. Through multiple sets of parameter ratio testing and comparison, the optimal network structure parameters suitable for the safflower filament detection task are screened to achieve model parameter optimization.

The Starnet_s050 backbone network adopts a four-stage cascaded feature extraction architecture. The network is composed of four feature extraction stages—Stage1, Stage2, Stage3, and Stage4—connected in series, with different stages undertaking differentiated feature learning tasks. The number of stacked modules after each stage directly determines the feature representation capability of that level. The original depth configuration parameters of this network are [1, 1, 3, 1], with a total stacking depth of 6. Its parameter allocation logic emphasizes enhancing deep semantic feature extraction in the third stage, which is suitable for conventional large-object and coarse-classification detection tasks. However, analysis in the context of the safflower filament detection scenario reveals that this original parameter configuration has obvious structural flaws: the difficulty in safflower filament detection does not lie in global semantic differences, but in the subtle distinctions in local texture, morphology, and compactness of the three classes. The original configuration overly stacks deep semantic modules in the third stage, causing redundancy of high-level features and wasted computation, while significantly weakening the extraction capability of mid-level structural features and detailed discriminative features in the second and third stages. This makes the model unable to effectively distinguish the three similar classes—mature, immature, and bud—leading to very easy classification confusion and false detection problems. To address this parameter adaptability shortcoming, this paper strictly maintains the total depth of the four stages of the Starnet backbone at 6, rearranges the module allocation ratio among stages, and designs seven comparative depth configuration schemes. The depth configurations of each scheme are shown in Table 3.

The remaining parameters of the models in the seven schemes are completely identical; only the depth distribution of the network layers is adjusted. Through uniform training and uniform evaluation metrics, the detection performance of the models under different parameter ratios is compared horizontally. Table 4 shows the performance of YOLOv11n-Starnet-ADown under different depth parameters.

In Table 4, *F*_1_ is the harmonic mean of precision and recall, calculated as follows:

(10) $$F_{1}=2\frac{P\cdot R}{P+R}$$

**Figure 5:**
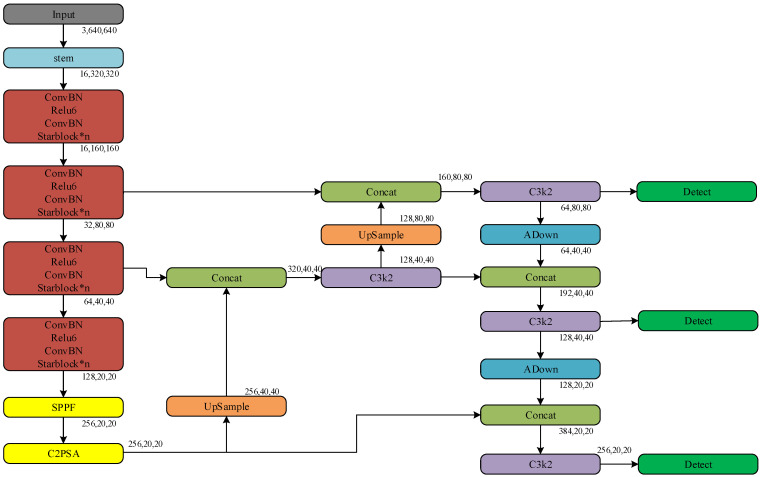
Structure of the YOLOv11n-Starnet-ADown model.

The final experimental results show that when the number of input channels for both the stem layer and stage1 layer is 16, and the depth configuration parameters are [1, 2, 2, 1], the model achieves the highest *F*_1_ score, indicating relatively optimal overall performance. Analyzing the reason: from the perspective of input channels, the first two layers of network parameters lose fewer features during feature extraction; the depth parameter configuration strengthens the number of stacked modules in the second and third feature stages of the network. These two stages are the core stages for extracting mid-level texture and morphological features of safflower filaments, and also the key levels for distinguishing the three classes of safflower filament targets. Moderately increasing the depth of the middle layers can enhance fine-grained feature learning capability and improve the discrimination accuracy of similar targets; while the shallow and deep layers maintain a lightweight configuration, effectively controlling the model memory footprint and computational cost, avoiding redundant parameters, and perfectly achieving the balance between lightweight design and high accuracy. The other configuration groups had issues such as insufficient shallow features, excessive high-level redundancy, and weak mid-level features, with detection accuracy and generalization ability inferior to the optimal configuration. The structure of the improved lightweight safflower detection model YOLOv11n-Starnet-ADown, based on YOLOv11n, is shown in Figure 5.

### 3.6. Model Evaluation Metrics

Model performance is evaluated using three metrics: precision (P), recall (R), and mean Average Precision at IoU 0.5 (mAP@0.5). Precision (P) represents the proportion of correctly identified positive samples among all detected positive samples. Recall (R) represents the proportion of positive samples that are correctly detected. Mean Average Precision (mAP@0.5) is used to characterize the detection and localization accuracy of safflower filaments. Based on the original design intention of the safflower harvesting robot, and on the premise of ensuring good overall precision, particular attention is paid to achieving high recall and localization accuracy for the harvestable mature safflower filament class.

The relevant formulas are as follows:

(11) $$P=\frac{\mathrm{TP}}{\mathrm{TP}+\mathrm{FP}}$$

(12) $$R=\frac{\mathrm{TP}}{\mathrm{TP}+\mathrm{FN}}$$

(13) $$\mathrm{mAP}=\frac{1}{N}\sum_{i=1}^{n}\int_{0}^{1}P(R)dR$$

where TP denotes the number of correctly predicted samples, FP denotes the number of samples predicted as positive but actually are negative, FN refers to the number of samples that are actually positive but predicted as negative, and N represents the total number of classes.

## 4. Experimental Results and Analysis

### 4.1. Experimental Conditions

The model training environment was set up on a workstation. The software environment configuration was as follows: operating system Windows 11, deep learning framework PyTorch 2.10.0, CUDA version 12.6, and Python 3.11.0 as the programming language. The hardware environment configuration was: NVIDIA A5000 GPU (24 GB VRAM), AMD Ryzen Threadripper PRO 3975WX processor, and 384 GB of RAM. All input images were uniformly resized to 640 × 640 pixels. The SGD optimizer was used with an initial learning rate of 0.0001 and a batch size of 64. The models were trained for 300 epochs, and both the best-performing model during training and the final model were recorded. The final model performance was evaluated using the best-performing model.

### 4.2. Performance Comparison of Different Loss Functions

To verify the performance advantage of the CIoU loss function for safflower recognition on the YOLOv11n-Starnet-ADown model, comparative experiments were conducted using four commonly used loss functions: DIoU, EIoU, GIoU, and ShapeIoU. The experimental results are shown in Table 5.

Analysis of the comparative experimental results in Table 5 reveals that the choice of loss function has a relatively pronounced effect on the training results of the YOLOv11n-Starnet-ADown model. In terms of precision, the CIoU and GIoU loss functions exhibit similar performance; the precision of the model using the CIoU loss function is 3%, 2.4%, and 3.8% higher than that of the models using DIoU, EIoU, and ShapeIoU loss functions, respectively. In terms of recall, the model using CIoU achieves a recall 1.5% higher than that of the model using GIoU. However, for the mAP@0.5 metric, the model using CIoU is merely 0.3% lower than the one using GIoU. This indicates that the CIoU loss function exhibits favorable performance on the safflower detection model YOLOv11n-Starnet-ADown.

### 4.3. Ablation Experiments and Comparative Experiments

#### 4.3.1. Ablation Experiments on the Improved Model

To evaluate the effectiveness of the proposed improvements to the YOLOv11n model, ablation experiments were conducted on the self-built safflower dataset using the YOLOv11n model as the baseline, under the previously described configuration environment and with identical training parameters. The experimental results are shown in Table 6, where A denotes the use of the Starnet backbone, B denotes the use of the ADown module in the neck network downsampling layer, and C denotes the use of optimized depth parameters for the Starnet backbone.

Analysis of Table 6 reveals that introducing only the Starnet backbone (YOLOv11n + A) reduces model size from 5.22 MB to 3.96 MB compared with the original YOLOv11n, achieving a significant lightweight improvement. However, the precision, mAP@0.5, and AP for all three flowering-stage targets decline notably; in particular, the AP for immature safflower drops sharply from 83.6% to 72.7%, and only the recall shows a slight increase. This indicates that simply replacing the backbone with Starnet compromises feature extraction capability, leading to a substantial degradation in detection accuracy for the small and less distinguishable immature safflower. Introducing only the ADown neck downsampling module (YOLOv11n + B) achieves lightweighting while simultaneously improving accuracy across all dimensions: precision reaches 92.1%, recall 90.5%, and mAP@0.5 hits 93.3%, the highest among all experimental groups. The detection accuracy for mature, bud, and immature safflower all improves synchronously, demonstrating that the ADown module optimizes the multi-scale feature downsampling process, reduces feature loss of tiny safflower flowers, and provides a positive gain for all three flowering-stage target classes, making it a core, effective improvement module.

Among the two-module combinations, both the YOLOv11n + A + C and YOLOv11n + A + B groups inherit the lightweight advantage brought by module A, further compressing memory usage. However, due to the shortcoming of the Starnet backbone in feature extraction, the detection accuracy for immature safflower remains lower than that of the original YOLOv11n model, and the recall and mAP metrics are inferior to those of the group improved with B alone. This suggests a feature matching conflict between the Starnet backbone and the standard downsampling structure, where combining them alone offsets the accuracy advantage of the ADown module. The three-module fusion model YOLOv11n + A + B + C is the target model of this experiment. By integrating the three improvements, its model size is reduced to 3.66 MB, the lowest across the entire experiment, representing a 29.9% reduction compared with the original YOLOv11n and achieving the optimal lightweight benefit. At the accuracy level, the advantages of each module complement and balance each other: precision at 91.6% is close to the baseline level, mAP@0.5 stabilizes at 92.0%, and the AP for mature and bud safflower reaches 96.5% and 94.2%, respectively, both outperforming the original model. Addressing the deficiency of module A, the AP for immature safflower detection recovers to 84.2%, surpassing the baseline of 83.6%. This proves that the Starnet backbone with optimized depth parameters and the ADown downsampling layer work synergistically, compensating for the insufficient feature extraction for tiny immature safflower when using the Starnet backbone alone.

The YOLOv11n + A + B + C model balances detection accuracy and deployment lightweight requirements. Compared with the original YOLOv11n, under the premise of significantly reducing memory consumption, the single-class detection accuracy for mature, bud, and immature safflower all improves without any loss in overall detection capability. It is better suited for deployment in low-compute-power scenarios such as embedded terminals and edge devices. This validates the feasibility and superiority of the integrated approach combining the lightweight Starnet backbone, the ADown downsampling module, and backbone depth parameter optimization for the safflower detection task.

#### 4.3.2. Comparative Experiments of Different Models

**Figure 6:**
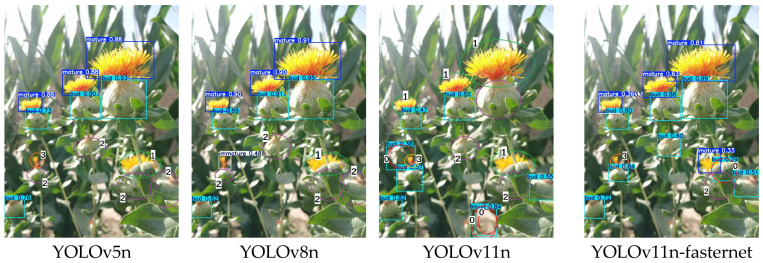

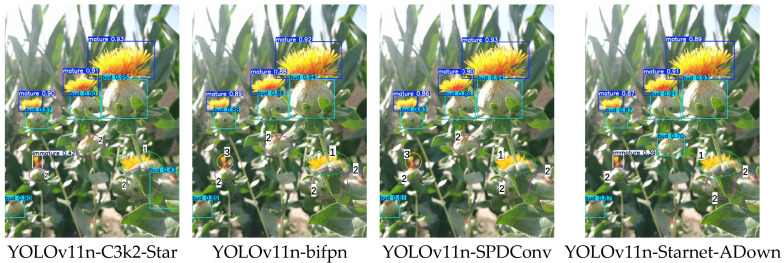
Comparison of detection results among different models.

To demonstrate the performance superiority of the proposed improved model, multiple models were selected for comparative experiments in terms of recognition accuracy, model size, and recognition speed. YOLOv5n, YOLOv8n, and YOLOv11n are lightweight baseline models of different versions, representing the classic lightweight architectures in the YOLO series. YOLOv11n-fasternet exemplifies the lightweight convolution replacement strategy, which reduces computational redundancy by introducing the lightweight convolution modules from FasterNet. YOLOv11n-C3k2-Star embodies the lightweight module embedding strategy, embedding StarBlock into the C3k2 residual structure to achieve localized lightweight enhancement. YOLOv11n-bifpn follows the feature pyramid optimization strategy, improving multi-scale feature fusion paths to boost accuracy. YOLOv11n-SPDConv adopts the improved downsampling strategy, utilizing SPD-Conv to address the loss of fine-grained information inherent in conventional downsampling operations. The experimental results are shown in Table 7. These results confirm the effectiveness of the YOLOv11n-Starnet-ADown model for safflower detection in natural unstructured environments. The model has a memory footprint of only 3.66 MB and achieves an average precision (AP) of 96.5% for the harvestable mature safflower filaments. It not only has the smallest model size but also exhibits excellent detection performance, with a clear advantage in comprehensive performance. The actual detection results are shown in Figure 6.

The YOLOv11n-Starnet-ADown model was compared in a multi-dimensional manner with YOLOv5n, YOLOv8n, the baseline YOLOv11n, and several improved YOLOv11n variants. The results in Table 7 indicate that in terms of detection accuracy, the precision of YOLOv11n-Starnet-ADown reaches 91.6%, on par with YOLOv8n, significantly higher than YOLOv5n (89.1%), YOLOv11n (91.1%), YOLOv11n-fasternet (90.2%), YOLOv11n-bifpn (88.3%) and YOLOv11n-C3k2-Star (91.2%), and only lower than YOLOv11n-SPDConv (93.2%), thus maintaining high precision under lightweight improvements. Its mAP@0.5 is 92.0%, which, although slightly lower than YOLOv8n (93.7%) and YOLOv11n-SPDConv (93.6%), it exhibits little difference from the native YOLOv5n (92.1%), YOLOv11n (92.4%), and YOLOv11n-C3k2-Star (92.5%), with the precision loss confined to within 0.5%. For the core class in safflower filament detection—mature—the model’s AP for mature safflower is 96.5%, ranking second among all models, lower only than YOLOv11n-SPDConv and higher than the baseline YOLOv11n (96.2%). This indicates that its ability to detect harvestable safflower filaments has not significantly deteriorated due to lightweight improvements and meets the accuracy requirements of real detection tasks.

In terms of inference efficiency, the FPS of YOLOv11n-Starnet-ADown is significantly higher than that of YOLOv11n-C3k2-Star and YOLOv11n-bifpn, but lower than the baselines YOLOv5n, YOLOv8n, YOLOv11n, and YOLOv11n-fasternet. In terms of hardware resource usage, the model size of YOLOv11n-Starnet-ADown is only 3.66 MB, the lowest among all compared models. This represents a 29.9% reduction compared to the baseline YOLOv11n, a 38.6% reduction compared to YOLOv8n, and a 59.5% reduction compared to YOLOv11n-SPDConv. The extremely low model size means the model can be easily deployed on memory-constrained edge devices such as embedded terminals and edge computing boxes, avoiding deployment failures or operation lag due to insufficient memory. Overall, YOLOv11n-Starnet-ADown achieves an optimal balance among detection accuracy, inference speed, and model size. Its core class-mature detection performance is excellent, its inference speed meets real-time requirements, and its hardware resource usage is extremely low. This fully validates the model’s effectiveness and proves that, compared to other models, it is better suited for edge computing scenarios with limited memory and computational power, providing an efficient and feasible technical solution for real-time field detection of safflower.

In Figure 6, square detection boxes denote the model’s recognition results. Red circles indicate false positives (labeled as category “0”), green circles indicate missed detections of the harvestable mature class (labeled as category “1”), purple circles indicate missed detections of the bud class (labeled as category “2”), and orange circles indicate missed detections of the immature class (labeled as category “3”). Analyzing Figure 6 reveals that YOLOv5n and YOLOv8n missed 6 and 5 targets, respectively, in actual testing. By examining the characteristics of these targets, it can be concluded that both YOLOv5n and YOLOv8n exhibit insufficient detection capability when dealing with targets that have colors similar to the background, and YOLOv5n is inferior to YOLOv8n in detecting small targets. The YOLOv11n model had the most severe missed detections in the test, particularly showing insufficient detection capability for larger targets, and produced 3 false detections, all caused by light reflections. For images under such complex environments, its detection of mature class targets completely failed, reflecting the model’s inadequate ability to extract features such as image textures. The YOLOv11n-fasternet model demonstrated the best overall detection performance but still had 1 false detection and 2 missed detections. The analysis suggests that this is due to targets whose color is close to the background and that are occluded; additionally, all missed targets were small, indicating the model’s limited small-target detection capability. The YOLOv11n-C3k2-Star, YOLOv11n-bifpn, and YOLOv11n-SPDConv models exhibited more missed detections in the test; they were strong at detecting unobstructed and larger targets but performed poorly on occluded targets. Furthermore, YOLOv11n-bifpn and YOLOv11n-SPDConv showed similar performance, both underperforming in detecting small immature class targets. The YOLOv11n-Starnet-ADown model did not perform well on occluded targets and those similar in color to the background but exhibited good detection performance on both large and small unobstructed targets, and the test results demonstrated excellent object localization accuracy.

### 4.4. Comparative Experiments on Detection Performance in Different Scenarios

To comprehensively and objectively evaluate the overall performance of the proposed model under complex real-world conditions, this study selects four mainstream deep learning object detection models—YOLOv5n, YOLOv8n, YOLOv11n, and YOLOv11n-Starnet—along with the proposed YOLOv11n-Starnet-ADown model for multi-scenario comparative experiments. By constructing differentiated test sample sets that simulate real environments with varying safflower maturity stages, front lighting, backlighting, occlusion, sunny conditions, and overcast conditions, the detection performance of the different models is quantitatively analyzed from multiple dimensions, including precision, recall, and mAP@0.5. The results are annotated on the detection effect images, where square detection boxes denote the model’s recognition results. Red circles indicate false positives (labeled as category “0”), green circles indicate missed detections of the harvestable mature class (labeled as category “1”), purple circles indicate missed detections of the bud class (labeled as category “2”), and orange circles indicate missed detections of the immature class (labeled as category “3”).

#### 4.4.1. Comparative Experiment on Detection Performance for Safflower Filaments at Different Maturity Stages

**Figure 7:**
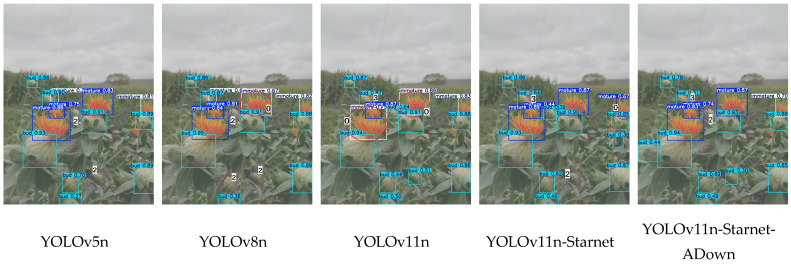
Comparison of detection effects at different levels of maturity.

Using the harvestable mature class and the immature class as control variables, the detection performance of the models under different maturity stages was tested. The test results are shown in Table 8, and the detection effects are illustrated in Figure 7.

Analysis of Table 8 shows that for mature safflower filaments, the overall detection accuracy of all models is at a high level. YOLOv11n-Starnet achieves the highest precision of 97.1%, while the target model YOLOv11n-Starnet-ADown achieves a precision of 96.5%, only slightly lower than the model incorporating only the Starnet improvement and superior to YOLOv11n (96.2%), YOLOv8n (95.7%), and YOLOv5n (95.3%). This demonstrates that both the Starnet backbone and the ADown module can effectively extract features of mature safflower filaments. The recall of the YOLOv11n-Starnet-ADown model is 95.2%, indicating stable missed-detection control. The mAP values of the five models differ very little, ranging only from 97.3% to 97.8%. The target model’s mAP@0.5 is 97.3%, only marginally lower than those of YOLOv5n, YOLOv8n, the original YOLOv11n, and YOLOv11n-Starnet. This indicates that for mature safflower filaments, adding the ADown downsampling module results in a negligible loss in overall average precision, and the model still possesses stable and reliable detection capability for mature samples. In the challenging detection scenario of immature safflower filaments, the detection performance of the YOLOv11n-Starnet-ADown model is relatively weaker; its detection precision is 1.8% and 0.9% lower than that of YOLOv8n and YOLOv11n-Starnet, respectively. The recall is 4.3%, 6.3%, and 4.3% lower than that of YOLOv5n, YOLOv8n, and YOLOv11n, respectively. The mAP@0.5 is 3.9%, 0.7%, and 1.5% lower than that of YOLOv8n, YOLOv11n, and YOLOv11n-Starnet, respectively. From the analysis of the relatively low recall rates of both the YOLOv11n-Starnet and YOLOv11n-Starnet-ADown models, the primary cause is attributed to missed detections. However, considering that safflower harvesting primarily focuses on the detection of mature safflower filaments, the overall performance of the YOLOv11n-Starnet-ADown model meets the requirements of safflower harvesting.

As can be seen in Figure 7, the YOLOv8n, YOLOv11n, and YOLOv11n-Starnet models all incorrectly detected mature class targets as immature class targets. The YOLOv5n and YOLOv11n-Starnet-ADown models correctly identified all mature class targets, but the YOLOv11n, YOLOv11n-Starnet, and YOLOv11n-Starnet-ADown models all missed detections of immature class targets.

#### 4.4.2. Comparative Experiment on Detection Performance Under Different Lighting Conditions

**Figure 8:**
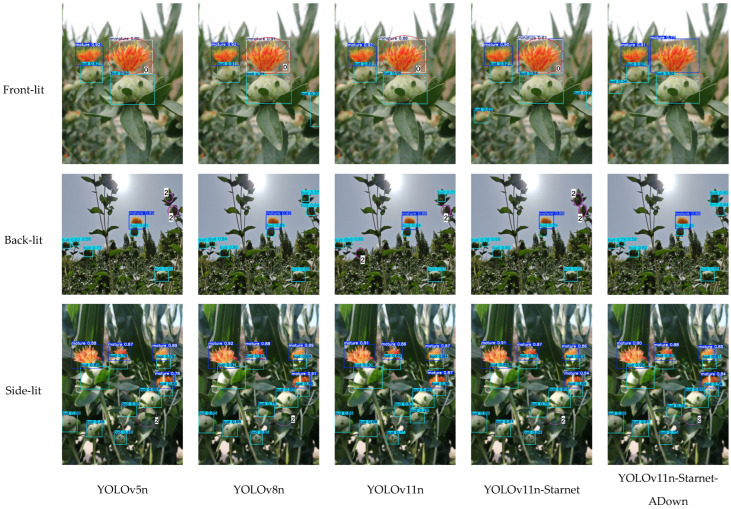
Detection effect under different lighting conditions.

To verify the practicability and robustness of the improved model under different lighting conditions in real safflower cultivation environments, front-lit, backlit, and side-lit conditions were used as control variables to test the detection performance of the five models. The test results are shown in Table 9, and the detection effects are illustrated in Figure 8.

Analysis of Table 9 shows that front-lit conditions, with sufficient illumination and clear target features, constitute an ideal operating scenario, and the overall accuracy of all models was in a high range. The proposed YOLOv11n-Starnet-ADown model achieved the highest mAP@0.5 of the entire group at 92.3%, with a balanced precision of 89.6% and recall of 89.2%, demonstrating no obvious weaknesses. This proves that the Starnet backbone combined with the ADown downsampling module can fully extract multi-scale safflower features under clear lighting, and its detection capability in conventional field front-lit scenarios is significantly superior to the other compared models.

Backlit conditions represent the most challenging scenario for safflower detection. Strong background light can easily blur target contours and reduce the contrast between foreground and background, causing the accuracy of all models to drop significantly; thus, this is a key scenario for testing the models’ anti-interference capability. YOLOv8n, YOLOv11n, and YOLOv11n-Starnet were the most severely affected by backlighting, with mAP@0.5 plunging to 61.8%, 66.4%, and 62.3%, respectively, and recall generally below 65%. YOLOv5n performed relatively stably with an mAP@0.5 of 66.9%, whereas the YOLOv11n-Starnet-ADown model reached an mAP@0.5 of 67.7%, the highest among all models in the backlit group. Its recall of 64.9% was essentially on par with the original YOLOv11n, and its precision of 74% surpassed those of the original YOLOv11n and YOLOv8n. This indicates that the ADown module optimizes multi-scale feature fusion and the downsampling process, alleviating the problem of feature loss for tiny safflower flowers under backlighting. In conjunction with the lightweight Starnet backbone, it effectively enhances the model’s ability to capture features of low-contrast backlit targets, achieving optimal robustness against backlight interference.

Under side-lit conditions, where light distribution is uneven and both unidirectional illumination and partial shadows coexist on safflower plants, the performance gaps among the models are relatively moderate. YOLOv11n-Starnet, leveraging its lightweight backbone advantage, achieved the highest mAP@0.5 of 89.6%. The proposed YOLOv11n-Starnet-ADown model recorded an mAP@0.5 of 88.5%, slightly lower than YOLOv11n-Starnet and YOLOv5n but higher than YOLOv8n and the original YOLOv11n. Its precision of 86.3% and recall of 84.4% remained stable, showing only a slight decline compared to the purely Starnet-based model without a precipitous drop in accuracy. Under the uneven illumination of side-lit conditions, the YOLOv11n-Starnet-ADown model still delivers reliable recognition performance.

As can be seen in Figure 8, under front-lit conditions, all models were able to accurately localize the targets; however, YOLOv5n, YOLOv8n, YOLOv11n, and YOLOv11n-Starnet all misdetected mature class targets as immature class targets. The YOLOv11n-Starnet-ADown model correctly identified all mature class targets. In addition, YOLOv8n, YOLOv11n-Starnet, and YOLOv11n-Starnet-ADown detected some small targets. Under backlit and side-lit conditions, safflower filaments had a distinct color difference from their surroundings and were easily detected correctly by all models. Due to shadows caused by the light incidence angle, the performance differences among the models were mainly concentrated in the detection of buds.

#### 4.4.3. Comparative Experiment on Detection Performance Under Occlusion

**Figure 9:**
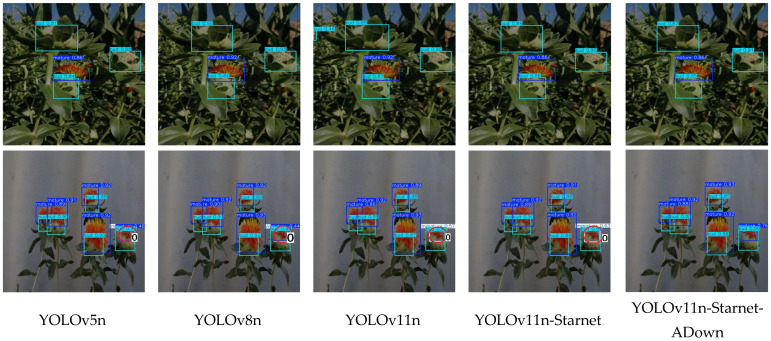
Detection effect under Occlusion.

In real harvesting environments, safflower plants are subject to occlusion by leaves, branches, and overlapping flowers. The test results of model detection performance under occlusion are shown in Table 10, and the detection effects are illustrated in Figure 9.

Analysis of Table 10 shows that YOLOv5n achieved the highest recall among all models at 95.1%, but its precision was only 85.6%, making it prone to many false detections, and its mAP@0.5 was slightly lower at 90.8%. YOLOv8n exhibited relatively balanced precision and recall, with an mAP@0.5 of 91.6%. The original YOLOv11n achieved a precision of 88.8%, superior to the former two, but its recall of 91.2% was low, indicating a significant missed detection problem for partially occluded safflower, as the downsampling process of the original network tends to lose the subtle features of incomplete filaments. The YOLOv11n-Starnet model, which only replaces the backbone with Starnet, achieved the highest mAP@0.5 of 92.3% among all models, demonstrating that the lightweight Starnet backbone can enhance the extraction of local, partially missing features. However, its recall was only 90.3%, indicating that optimizing the backbone alone with a lightweight design cannot effectively ameliorate the missed detection deficiency for occluded targets. The YOLOv11n-Starnet-ADown model, integrating the Starnet backbone with the ADown downsampling module, achieved balanced optimization of accuracy metrics. Its recall increased to 92.3%, a 2.0 percentage point improvement over YOLOv11n-Starnet, substantially alleviating missed detections of partially occluded safflower. Although its precision decreased slightly to 86.2%, it was still higher than that of YOLOv5n, demonstrating good false detection control. The overall mAP@0.5 was stable at 91.9%, only slightly lower than YOLOv11n-Starnet, with a minimal gap between its comprehensive detection accuracy and that of the best single-modification model. This result indicates that the ADown downsampling module optimizes the multi-scale feature transmission pathway, preserving edge details of locally occluded filaments during the downsampling stage, thereby compensating for the insufficient recall capability of the Starnet backbone and achieving a balance between precision and recall.

Analysis of Figure 9 shows that all models were able to detect the targets reasonably well in actual detection; the main differences lay in target classification. In the detection of some images, the four models—YOLOv5n, YOLOv8n, YOLOv11n, and YOLOv11n-Starnet—exhibited false detections.

#### 4.4.4. Comparative Experiment on Detection Performance Under Different Weather Conditions

**Figure 10:**
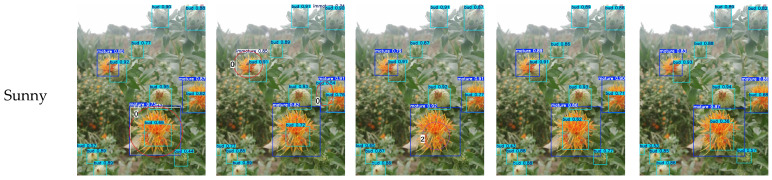

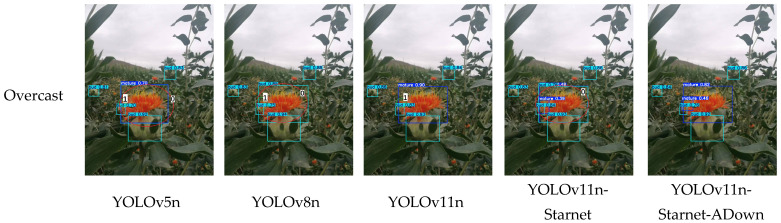
Detection effects under different weather conditions.

Different weather conditions affect the contrast and color saturation of captured images, thereby impacting model detection. The performance of the models was tested under sunny and overcast conditions, respectively. The test results are shown in Table 11, and the detection effects are illustrated in Figure 10.

Under sunny conditions with sufficient light, the texture details of safflower are clear, and the accuracy of all models is generally in a high range. The proposed YOLOv11n-Starnet-ADown model was roughly on par with the other models in terms of recall, but its precision and mAP@0.5 were lower than those of the other models, indicating that the strong light in sunny environments has a relatively large impact on its recognition performance. Under overcast conditions with dim light and no strong reflections in the images, YOLOv11n-Starnet-ADown demonstrated significantly superior performance compared with the other four models. The mAP@0.5 values of YOLOv5n, YOLOv8n, YOLOv11n, and YOLOv11n-Starnet were 89.0%, 89.2%, 86.6%, and 87.7%, respectively, with recall rates all below 84%; a large number of safflower targets were missed under low light. In contrast, YOLOv11n-Starnet-ADown achieved a comprehensive performance comeback under overcast conditions, with precision reaching the highest among all models at 91.4%, recall increasing to 86.0%, and mAP@0.5 as high as 91.4%, significantly outperforming the other compared models. This indicates that the ADown downsampling module can preserve the faint contours and filament details of safflower during the downsampling process under low light, forming a synergistic effect with the lightweight Starnet backbone, and substantially enhancing the model’s ability to capture blurred targets in low-illumination conditions.

As can be seen in Figure 10, under sunny conditions, YOLOv5n produced duplicate detections, YOLOv5n and YOLOv8n exhibited false detections, and YOLOv11n missed detections. Both YOLOv11n-Starnet and YOLOv11n-Starnet-ADown demonstrated relatively good detection performance. Under overcast conditions, YOLOv5n, YOLOv8n, and YOLOv11n-Starnet exhibited false detections; YOLOv5n and YOLOv11n-Starnet produced duplicate detections; YOLOv5n, YOLOv8n, and YOLOv11n missed small targets; and the YOLOv11n-Starnet-ADown model exhibited good detection performance.

In summary, compared with the other four models, the YOLOv11n-Starnet-ADown model exhibits better detection performance in diverse real-world environments, with fewer false and missed detections. It is adaptable to all-day operation scenarios with alternating sunny and overcast skies, branch and leaf occlusion, and varying light incidence angles. The proposed model shows significant advantages in detection accuracy, anti-interference capability, and environmental adaptability.

## 5. Discussion

Aiming at the performance requirements of detection models for intelligent safflower harvesting—namely low computational cost and high detection accuracy—this paper proposes a lightweight network, YOLOv11n-Starnet-ADown, based on the YOLOv11n model, through baseline model screening, lightweight module performance comparison, and backbone network layer parameter optimization. The Starnet network is selected as the backbone, which can significantly reduce the number of model parameters and memory footprint, making the model suitable for edge computing scenarios in intelligent safflower harvesting. To address the issue of the Starnet network’s relatively weak image feature extraction capability, the ADown module is introduced into the neck network of the model to enhance its small-object detection ability and further reduce the number of model parameters. In view of the still unsatisfactory performance of the constructed model, an attempt was made to adjust the depth parameters of each stage in the Starnet backbone. After multiple experiments, an optimal set of network depth parameters was obtained. Through ablation experiments and multi-model comparative experiments, the applicability of the YOLOv11n-Starnet-ADown model in safflower recognition was verified.

During model construction, an attempt was made to set the number of output channels of the stem layer in the Starnet backbone to the native value of 32, while keeping the number of output channels of stage1 at 16. However, the experimental results showed that the model’s size increased without any improvement in training accuracy. The analysis suggests that when the input image size is (3, 640, 640), with the stem layer output channels set to 32 and stage1 output channels set to 16, the output shapes of the stem and stage1 layers are (32, 320, 320) and (16, 160, 160), respectively. The drastic change in feature maps between the two layers causes considerable feature loss, ultimately leading to poor model performance. If the number of output channels of stage1 were also set to 32, the model’s computational cost and model size would increase, which does not align with the objectives of this study. In this paper, the ADown module is integrated only into the neck network of the model to replace traditional downsampling operations; it is not applied to the backbone network. The YOLOv11n-SPDConv model has demonstrated good detection performance for small objects [28], and in the experiments of this paper, it also exhibited excellent detection performance, but its computational cost and model size are excessively large.

The core design philosophy of the Starnet backbone is to achieve high-dimensional feature representation in a low-dimensional space through star operations. Its structure—comprising DWConv + BN + dual fully connected layers—pursues extreme channel compression and computational efficiency. In contrast, the ADown module relies on average pooling preprocessing, channel splitting, and a dual-path parallel downsampling mechanism, which performs optimally only when fed with relatively rich multi-scale information. When the low-dimensional feature maps output by Starnet are directly fed into the ADown module, the insufficient number of feature channels limits the feature diversity after ADown’s dual-path splitting, preventing the module from fully unleashing its advantage in retaining fine details of small targets. Moreover, Starnet’s lightweight design already sacrifices some spatial details during downsampling; although ADown can partially compensate for this deficiency, it cannot fully restore the edge and texture information lost from the original high-resolution features. In particular, strong sunlight in clear-sky conditions can cause local overexposure and reduced contrast in safflower images, and the lightweight channel compression mechanism of the Starnet backbone further exacerbates the loss of detail in highlight regions, while ADown’s reliance on average pooling preprocessing tends to over-smooth both foreground and background information under intense light, thereby weakening the discriminability between targets and background.

It should be noted that the lightweight improvements in this paper have so far been validated only at the algorithmic level—that is, indirectly demonstrated through metrics such as model file size (3.66 MB), theoretical computational cost (4.6 × 10^11^ FLOPs), and inference speed (200 FPS, measured on an NVIDIA A5000 workstation). When deployed on edge devices with significantly reduced computational power (e.g., the Jetson Nano, with a theoretical peak of approximately 0.5 TFLOPS, about 1/50 that of the A5000), linear scaling would still yield over 4 FPS; with TensorRT acceleration (typically providing a 2–3× speedup), it could potentially reach 10–15 FPS, which would essentially meet the real-time harvesting requirements for low-density safflower planting scenarios. However, due to current research constraints, systematic deployment testing on typical embedded edge devices such as Jetson Nano and RK3588 has not yet been conducted, and actual hardware measurement data on inference latency, power consumption, and memory usage are not available. Therefore, the deployment feasibility and real-time performance of the proposed model on actual harvesting robot terminals still require further hardware verification, which constitutes a key direction for our future work.

The model built in this paper performs well on the test set; however, limited by the size of the dataset and sample diversity, the model may have a certain risk of overfitting and may fail to learn safflower features across all real scenarios. This aspect is also reflected in the performance comparison between the proposed model and the other four conventional models. Under bright sunny conditions, the detection accuracy degrades (mAP@0.5 of 87.2%, compared with 90.8% for YOLOv11n-Starnet), and further improvement is needed through illumination-adaptive strategies. In addition, the model’s recognition accuracy is suboptimal under sunny conditions, resulting in a higher number of false negative (*FN*) detections, which may be the primary cause for the relatively low recall of the target model in Table 6. Future research will employ larger datasets with richer sample diversity for training and validation and introduce more rigorous cross-validation strategies to quantitatively evaluate the degree of model overfitting.

In future studies, the safflower dataset will be enriched with image data under more common conditions to strengthen the model’s feature learning capability for both backlit and strong-light tasks. The accuracy and real-time performance of safflower filament detection and localization will be further optimized to advance the implementation of safflower detection, localization, and harvesting solutions. Through continuous iterative optimization in the above directions, it is expected that more advanced lightweight models will be developed, which is anticipated to substantially enhance the engineering practical value and academic influence of the algorithm proposed in this paper, thereby providing technical support for intelligent safflower harvesting.

## 6. Conclusions

(1) Aiming at the requirements of low missed detection rate, high accuracy, and low computational cost for recognition models deployed on safflower harvesting equipment, this paper proposes an improved model, YOLOv11n-Starnet-ADown, based on the YOLOv11n model. This model uses the Starnet network to replace the backbone of YOLOv11n and uses ADown to replace the downsampling module in the neck network of YOLOv11n. Without reducing the detection capability of the model, it reduces the size of the model. The depth parameters of the Starnet backbone are optimized by increasing the depth of the middle layers, making the model more suitable for feature extraction of safflower images and enhancing its learning capability. Several loss functions were compared, and the CIoU loss function was selected as being more suitable for the safflower detection task, further improving the detection accuracy of the model. A safflower dataset was constructed by capturing images of safflower in natural environments and applying image augmentation, which was then used for model training and testing.
(2) The ablation experiment results show that the proposed YOLOv11n-Starnet-ADown model possesses comprehensive performance advantages. Its precision, recall, and mean Average Precision reach 91.6%, 87.8%, and 92%, respectively. Compared with the relevant metrics of the baseline YOLOv11n model, precision increased by 0.5%, while recall and mean Average Precision decreased by 2.1% and 0.4%, respectively. However, the mean Average Precision for the main class—harvestable mature safflower—reached 96.5%, which is 0.3% higher than that of the YOLOv11n model. The overall model size is only 3.66 MB, a reduction of 29.9% compared with the YOLOv11n model, indicating that the proposed improved model possesses core detection capability comparable to that of YOLOv11n.
(3) Comparative tests were conducted among different detection models. The results demonstrate that the YOLOv11n-Starnet-ADown model achieves good performance metrics on the safflower dataset in terms of overall recognition precision, recall, mAP@0.5, and model memory footprint, with excellent recognition accuracy for the harvestable mature safflower filament targets. Furthermore, under complex scenarios involving varying maturity levels, side lighting, front lighting, occlusion, and overcast conditions, the improved model consistently exhibits distinct advantages and high robustness.

## Figures and Tables

**Table 1:** Statistics of each category in the safflower dataset.

Category	Mature	Bud	Immature
Count	12,306	29,021	1156

**Table 2:** Comparison of baseline model performance.

Model	Precision P (%)	Recall R (%)	mAP@0.5 (%)	Model Size (MB)
YOLOv11n-C3k2-Faster	89.9	90.5	91.2	4.65
YOLOv11n-C3k2-Star	91.2	89.4	92.5	5.05
YOLOv11n-ADown	92.1	90.5	93.3	4.33
YOLOv11n-bifpn	88.3	89.8	92.1	4.00
YOLOv11n-Starnet	85.8	90.5	91.2	3.96

**Table 3:** Network depth distribution of different schemes for the Starnet backbone.

Scheme	Stage1	Stage2	Stage3	Stage4
1	1	1	3	1
2	2	2	1	1
3	2	1	2	1
4	1	2	1	2
5	2	1	1	2
6	1	1	2	2
7	1	2	2	1

**Table 4:** Performance of the YOLOv11n-Starnet-ADown model with different depth parameters.

Scheme	Precision P (%)	Recall R (%)	mAP@0.5 (%)	Model Size (MB)	F1
1	88.5	88.9	91.9	3.79	0.887
2	89.0	87.7	91.2	3.59	0.883
3	88.1	87.7	91.5	3.65	0.879
4	87.3	88.5	91.6	3.89	0.879
5	91.9	86.8	91.6	3.87	0.893
6	89.6	88.6	92.3	3.95	0.891
7	91.6	87.8	92.0	3.66	0.897

**Table 5:** Performance comparison of models with different loss functions.

Model	P (%)	R (%)	mAP@0.5 (%)
YOLOv11n-Starnet-ADown + DIoU	88.6	88.6	91.7
YOLOv11n-Starnet-ADown + EIoU	89.2	90.7	91.1
YOLOv11n-Starnet-ADown + GIoU	92.0	86.3	92.3
YOLOv11n-Starnet-ADown + ShapeIoU	87.8	87.9	91.9
YOLOv11n-Starnet-ADown + CIoU	91.6	87.8	92.0

**Table 6:** Ablation experiment results.

Model	P (%)	R (%)	mAP@0.5 (%)	AP (%)			Model Size (MB)
				Mature	Bud	Immature	
YOLOv11n	91.1	89.9	92.4	96.2	93.5	83.6	5.22
YOLOv11n + A	85.8	90.5	91.2	94.4	90.4	72.7	3.96
YOLOv11n + B	92.1	90.5	93.3	97.2	94.7	84.5	4.33
YOLOv11n + A + C	88.9	89.3	91.2	96.4	93.3	77.1	3.91
YOLOv11n + A + B	88.5	88.9	91.9	96.2	93.1	76.2	3.79
YOLOv11n + A + B + C	91.6	87.8	92.0	96.5	94.2	84.2	3.66

**Table 7:** Comparison of safflower detection performance of different models.

Model	P (%)	R (%)	mAP@0.5 (%)	AP(%)			FPS (s^−1^)	Memory Cost (MB)
				Mature	Bud	Immature		
YOLOv5n	89.1	90.6	92.1	95.3	91.8	80.3	250	5.02
YOLOv8n	91.6	91.4	93.7	95.7	93.1	86.0	312	5.96
YOLOv11n	91.1	89.9	92.4	96.2	93.5	83.6	303	5.22
YOLOv11n-fasternet	90.2	90.6	92.3	96.1	93.2	81.3	385	7.74
YOLOv11n-C3k2-Star	91.2	89.4	92.5	95.8	93.3	84.6	159	5.05
YOLOv11n-bifpn	88.3	89.8	92.1	95.5	92.6	76.6	175	4.0
YOLOv11n-SPDConv	93.2	89.7	93.6	97.5	94.6	87.6	278	9.04
YOLOv11n-Starnet-ADown	91.6	87.8	92.0	96.5	94.2	84.2	200	3.66

**Table 8:** Detection results at different maturity stages.

Maturity	Model	P (%)	R (%)	mAP@0.5 (%)
Mature	YOLOv5n	95.3	95.6	97.6
	YOLOv8n	95.7	96.2	97.8
	YOLOv11n	96.2	95.6	97.5
	YOLOv11n-Starnet	97.1	95.0	97.4
	YOLOv11n-Starnet-ADown	96.5	95.2	97.3
Immature	YOLOv5n	80.3	81.4	82.3
	YOLOv8n	86.0	83.4	86.5
	YOLOv11n	83.6	81.4	83.3
	YOLOv11n-Starnet	85.1	72.5	84.1
	YOLOv11n-Starnet-ADown	84.2	77.1	82.6

**Table 9:** Detection results under different lighting conditions.

Lighting Condition	Model	P (%)	R (%)	mAP@0.5 (%)
Front-lit	YOLOv5n	86.8	84.9	88.9
	YOLOv8n	89.1	86.5	90.1
	YOLOv11n	89.1	82.4	87.4
	YOLOv11n-Starnet	91.1	85.5	90.8
	YOLOv11n-Starnet-ADown	89.6	89.2	92.3
Back-lit	YOLOv5n	84.6	59.3	66.9
	YOLOv8n	72.0	54.7	61.8
	YOLOv11n	71.6	64.8	66.4
	YOLOv11n-Starnet	78.6	50.6	62.3
	YOLOv11n-Starnet-ADown	74.0	64.9	67.7
Side-lit	YOLOv5n	85.9	88.4	88.9
	YOLOv8n	82.6	90.9	86.8
	YOLOv11n	84.9	88.4	86.2
	YOLOv11n-Starnet	87.3	86.3	89.6
	YOLOv11n-Starnet-ADown	86.3	84.4	88.5

**Table 10:** Detection results under occlusion.

Model	P (%)	R (%)	mAP@0.5 (%)
YOLOv5n	85.6	95.1	90.8
YOLOv8n	87.7	94.0	91.6
YOLOv11n	88.8	91.2	91.7
YOLOv11n-Starnet	88.6	90.3	92.3
YOLOv11n-Starnet-ADown	86.2	92.3	91.9

**Table 11:** Detection results under different weather conditions.

Lighting Condition	Model	P (%)	R (%)	mAP@0.5 (%)
Sunny	YOLOv5n	84.3	89.1	89.6
	YOLOv8n	87.0	91.8	89.1
	YOLOv11n	89.0	87.4	88.8
	YOLOv11n-Starnet	89.4	86.7	90.8
	YOLOv11n-Starnet-ADown	84.2	89.0	87.2
Overcast	YOLOv5n	91.0	83.3	89.0
	YOLOv8n	87.2	83.9	89.2
	YOLOv11n	89.3	82.8	86.6
	YOLOv11n-Starnet	90.5	82.4	87.7
	YOLOv11n-Starnet-ADown	91.4	86.0	91.4

## Data Availability

The data that support the findings of this study are available within the manuscript.
